# CGRP Reduces Apoptosis of DRG Cells Induced by High-Glucose Oxidative Stress Injury through PI3K/AKT Induction of Heme Oxygenase-1 and Nrf-2 Expression

**DOI:** 10.1155/2019/2053149

**Published:** 2019-11-25

**Authors:** YaDong Liu, SiCong Zhang, Jun Xue, ZhongQing Wei, Ping Ao, BaiXin Shen, LiuCheng Ding

**Affiliations:** ^1^Department of Urology, The Second Affiliated Hospital of Nanjing Medical University, Nanjing, Jiangsu Province, China; ^2^Department of Urology, The Third People's Hospital of Yancheng, Yancheng, Jiangsu Province, China

## Abstract

Dorsal root ganglion (DRG) neurons, which are sensitive to oxidative stress due to their anatomical and structural characteristics, play a complex role in the initiation and progression of diabetic bladder neuropathy. We investigated the hypothesis that the antioxidant and antiapoptotic effects of CGRP may be partly related to the expression of Nrf2 and HO-1, via the phosphatidylinositol 3-kinase (PI3K)/AKT pathway, thus reducing apoptosis and oxidative stress responses. This study shows that CGRP activates the PI3K/AKT pathway, thereby inducing increased expression of Nrf2 and HO-1 and resulting in the decrease of reactive oxygen species and malondialdehyde levels and reduced neuronal apoptosis. These effects were suppressed by LY294002, an inhibitor of the PI3K/AKT pathway. Therefore, regulation of Nrf2 and HO-1 expression by the PI3K/AKT pathway plays an important role in the regulation of the antioxidant and antiapoptotic responses in DRG cells in a high-glucose culture model.

## 1. Introduction

The prevalence of diabetes mellitus (DM) has significantly increased worldwide, accompanied by an increase in the incidence of obesity. Diabetic cystopathy (DCP) is one of the primary complications of DM in the lower urinary tract (LUT), and subjects often experience a series of symptoms, characterized by decreased bladder sensation, increased bladder capacity, impaired bladder contractility, and increased residual urine [1].

Multiple factors, including neuronal dysfunction, detrusor dysfunction, urothelial or urethral dysfunction, and polyuria, all contribute to the development of DCP [2, 3]. Dorsal root ganglia (DRGs) as a primary neuron had been confirmed to participate in the pathogenesis of diabetic bladder dysfunction [4]. However, the molecular mechanism leading to DCP in neuronal dysfunction remains largely unclear, although accumulating evidence shows that it is related to oxidative stress injury [5–7]. This has been confirmed by previous studies in diabetic rats treated with antioxidants [8, 9]. Meanwhile, various aspects of bladder function, including maximal bladder volume, bladder pressure, and maximal bladder pressure, measured by urodynamics, were partly improved. Bladder dysfunction due to neuronal dysfunction involves complex and sophisticated interactions among the somatic and autonomic afferent and efferent pathways. Some studies have reported a close relationship between diabetes-induced peripheral neuropathy and bladder dysfunction [10]. This has been further confirmed by neuromodulation in the treatment of voiding dysfunction in diabetic rats [11].

Nuclear factor-erythroid 2-related factor 2 (Nrf2) is a key transcription factor that regulates cellular redox homeostasis and has been confirmed to play a neuroprotective role in cerebral ischemia-reperfusion injury (CIRI) [12]. Heme oxygenase-1 (HO-1) is believed to participate in the process of heme catabolism, directly affecting the antioxidative balance in the body, and is also regulated by Nrf2 [13]. The PI3-kinase/AKT-mediated pathway is involved in antioxidant and antiapoptotic activities through Nrf2/HO-1 in mouse *β*-cells [14]. Recently, it has been reported that neurotrophic factors and neurotransmitters may be associated with the pathogenesis of diabetic bladder dysfunction and oxidative stress [15]. Calcitonin gene-related peptide (CGRP) is a 37-amino-acid-long regulatory peptide derived from the calcitonin gene located on chromosome 11 [16, 17]. We confirmed that the expression of CGRP in the dorsal root ganglia (DRGs) of the control group was significantly higher than that in the DM group (unpublished). Some studies have demonstrated the widespread expression and protective effect of CGRP in both neurons and cardiomyocytes [16, 18]. Meanwhile, the experiment suggested that CGRP plays a pivotal role in the regulation of apoptosis and oxidative stress via the PI3K/AKT pathway [18]. Thus, we speculated that the oxidative stress damage and apoptosis in neurons play a role in the pathogenesis of diabetic bladder dysfunction. However, the related mechanism of DRG injury in high-glucose conditions remains largely elusive.

The main objectives of our study were to demonstrate (1) the oxidative stress injury of DRGs under high-glucose conditions and (2) the neuroprotective effect of CGRP associated with increased expression of HO-1 and Nrf2 mediated by the PI3-kinase/AKT signaling pathway.

## 2. Materials and Methods

### 2.1. Animals and Treatment

Female Sprague-Dawley rats (4–5 weeks old; Nanjing Medical University Animal Laboratory, Nanjing, China) weighing 110 ± 10 g were provided by the Animal Laboratory of Nanjing Medical University and fed a standard rodent diet with access to water ad libitum. All protocols were performed in accordance with the guidelines of Jiangsu Province Animal Research Advisory Committee for the Care and Use of Laboratory Animals. The experiments were approved by the Ethics Committee of Nanjing Medical University. The rats were killed at the same hour of the day.

### 2.2. Isolation of DRG Cells

Sprague-Dawley rats were euthanized by cervical dislocation, and DRGs were aseptically collected from L3 to S3 spinal levels. All DRGs were minced into small pieces and digested with 0.25% trypsin (Sigma-Aldrich) (10 min) and 0.1% collagenase (Sigma-Aldrich) (3–5 min) in DMEM/Nutrient Mixture F12 (Gibco) at 37°C. After centrifugation, the cells were resuspended in DMEM/Nutrient Mixture F12 medium containing 2% B27 (Invitrogen, Carlsbad, CA) and 10 ng/mL NGF (Sigma-Aldrich, St. Louis, MO). The cells were seeded in 96-well plates (200 *μ*L per well), yielding a density of 5 × 10^4^ cells/well. The cells were cultured in a humid incubator at 37°C and 5% CO_2_.

### 2.3. Establishment of a DRG Cell Model

Cultured cells were exposed to 25, 45, 50, 100, 200, and 400 mmol/L of glucose following seeding for 24 h and 48 h. For the cell viability assays, the concentration of glucose was determined to be 45 mmol/L at 48 h. The DRGs were exposed to 45 mmol/L of glucose and treated with CGRP alone or CGRP+LY294002 (PI3K/AKT inhibitor, 10 *μ*M). All the above cultures were incubated at 37°C in a humidified 5% CO_2_ incubator. The oxidative stress index, which includes a measure of the reactive oxygen species (ROS), malondialdehyde (MDA), and superoxide dismutase (SOD) levels, was determined for the cultured cells.

### 2.4. Cell Viability Assay

The viability of DRG cells was determined using the CCK-8 assay (a common method for a cell viability assay). The CCK-8 kit was purchased from Beyotime Company (number C0040) and was strictly in accordance with the manufacturer's instructions mentioned. Briefly, 4 × 10^3^ DRG cells were plated in each well of a 96-well plate. Media (100 *μ*L) were added to each well. At 48 h after incubation, 10 *μ*L of the CCK-8 solution was added to each well and the plate was incubated for another 2 h. The optical density of each well was measured at 450 nm using a microplate reader.

### 2.5. Apoptosis Assay

Flow cytometric analysis was performed after annexin V-FITC labeling to evaluate cell apoptosis.

The cells pretreated with CGRP for 48 hours were trypsinized and centrifuged at 12,000 g for 3 min at 4°C, followed by washing with staining buffer, and resuspension in binding buffer. Cells were stained with annexin V-FITC, followed by the addition of propidium iodide. Samples were then analyzed for apoptotic cells using a FACScan instrument.

### 2.6. Biochemical Assessment

All the SOD (MM-0385R1, purchased from Shanghai Huyu Biotechnology Company) and MDA (MM-0386R1, purchased from Shanghai Huyu Biotechnology Company) experimental procedures were strictly in accordance with the manufacturer's instructions mentioned in the kits. The disrupted cell or tissue lysate was centrifuged at 12,000 g for 5 min, and the supernatant was mixed with the detection solution and incubated for 40 min at 95°C in a water bath. After cooling, the samples were centrifuged at 4,000 g for 10 min. The optical density values of each group were measured and recorded at 450 nm with a 1 cm light path.

### 2.7. Measurement of ROS Production

DRG cells were loaded with 5 *μ*mol/L DCFH-DA at 37°C for 30 min. The culture medium was removed, and the plates were washed three times with 0.1 mmol/L PBS (pH 7.4, Invitrogen) to remove the excess DCFH-DA. The fluorescence intensity of the oxidized derivative was analyzed using flow cytometry. The ROS values of the various treatment groups were calculated relative to the control cells.

### 2.8. Western Blotting

Western blotting was used to examine the expression of AKT, phosphorylated AKT (p-AKT), Nrf2, and HO-1. Total proteins (20 *μ*g) from each sample were electrophoresed on a 12% SDS-polyacrylamide gradient gel and transferred to nitrocellulose membranes (Millipore). The membrane was blocked with 5% fat-free milk in rinse buffer for 30 min and incubated for 2 h with the following primary antibodies: AKT antibody (1 : 1000, Proteintech), p-AKT antibody (1 : 1000, Santa Cruz Biotechnology), Nrf-2 (1 : 1000 Proteintech), HO-1 (1 : 500 Proteintech), and anti-*β*-catenin (1 : 1000, Abcam). Next, they were incubated with an HRP-conjugated secondary antibody (goat anti-rabbit IgG 1 : 5000, Beijing Zhongshan Golden Bridge Biotechnology Co. Ltd.) and visualized using the enhanced chemiluminescence (ECL) system (Pierce, Rockford, IL, USA). *β*-Actin was used as a reference protein.

### 2.9. Statistical Analysis

All data are presented as the mean ± standard error of the mean (SEM). The difference among groups was analyzed using one-way ANOVA, followed by two independent sample tests to compare the differences between the two groups, using the SPSS 22.0 software. Figures were constructed using GraphPad Prism 5 (GraphPad Software, San Diego, CA). *p* < 0.05 and *p* < 0.01 were considered to indicate statistically significant differences.

## 3. Results

### 3.1. Effect of Glucose Concentration on Cell Viability

**Figure 1:**
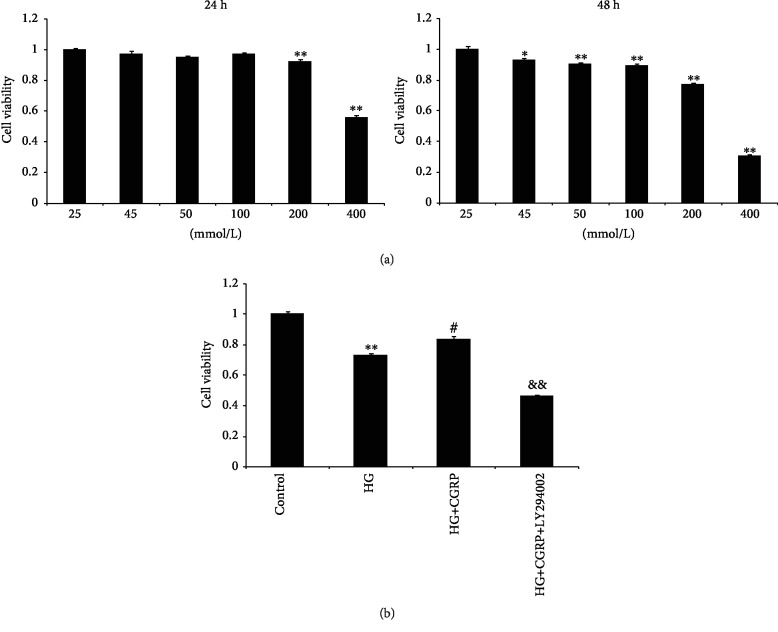
(a) High glucose inhibits DRG cell viability. DRG cell viability decreased in a dose-dependent manner with increasing concentrations of glucose. Dissociated rat DRG cells were cultured in different concentrations of glucose with 10 ng/mL NGF for 24 h and 48 h. The cell viability at 24 h with glucose concentrations of 200 mmol/L and 400 mmol/L was significantly decreased compared to the control (25 mmol/L). At 48 h, we found that the cell viability was significantly reduced at all glucose concentrations compared to the control. ^∗^*p* < 0.05, compared to the control; ^∗∗^*p* < 0.01, compared to the control. (b) Cell viability of DRG neurons in different groups after 48 h. Treatment with HG, HG+CGRP, and HG+CGRP+LY294002. ^∗∗^*p* < 0.01, compared to the control; ^#^*p* < 0.05, compared to the HG group; ^&&^*p* < 0.01, compared to the HG+CGRP group.

The CCK-8 assay was performed to determine the concentration range of glucose to be used. A glucose concentration lower than 200 mmol/L did not affect cell viability in 24 h. Next, we incubated the cells in the same condition and performed the CCK-8 assay after 48 h of incubation. Cell viability was reduced up to a glucose concentration of 45 mmol/L. The cell viability of DRG cells was reduced in a dose-dependent manner with increasing glucose (Figure 1(a)). Thus, we selected the moderate glucose concentration (45 mmol/L) as the high-glucose (HG) culture condition. This glucose concentration was similar to that used in previous studies [19, 20]. At the indicated glucose concentration, the cell viability of DRG cells in the HG+CGRP group was significantly improved compared to the HG group (*p* < 0.01). When pretreated with LY294002, the HG+CGRP+LY294002 group showed a marked decrease in cell viability compared to the HG+CGRP group (*p* < 0.01) (Figure 1(b)).

### 3.2. The Effect of CGRP on DRG Cells in Apoptosis

**Figure 2:**
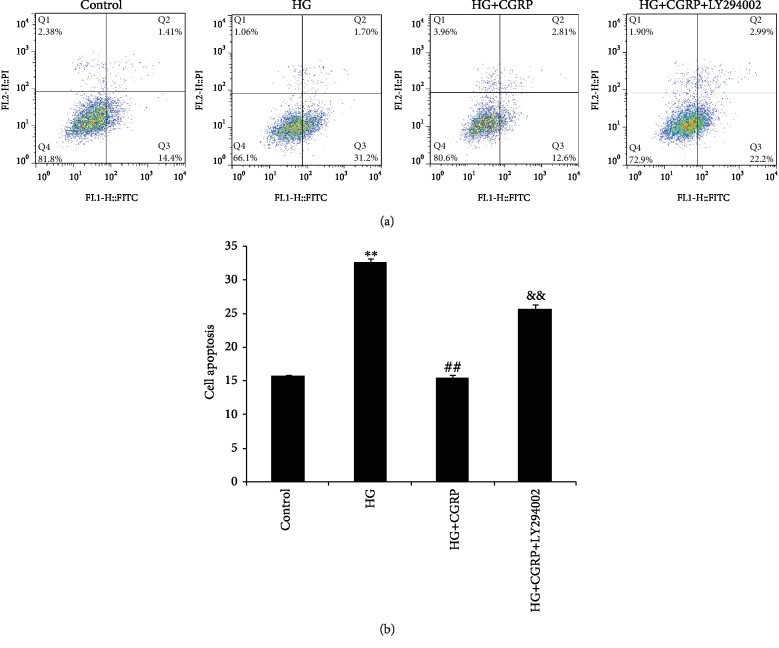
(a) After 48 h of treatment with HG, HG+CGRP, and HG+CGRP+LY294002, DRG cells were subjected to oxidative stress injury, and the relative number of annexin V-positive cells was determined using flow cytometry (b) Apoptosis of DRG neurons after HG, HG+CGRP, or HG+CGRP+LY294002 treatment for 48 h. ^∗∗^*p* < 0.01, compared to the control; ^##^*p* < 0.01, compared to the HG group; ^&&^*p* < 0.01, compared to the HG+CGRP group.

The apoptotic cell numbers for each group are shown in Figures 2(a) and 2(b). It was observed that the apoptosis of DRG cells in a high-glucose medium was significantly increased as compared to the control group (*p* < 0.01), and then it decreased after CGRP treatment (*p* < 0.01). When pretreated with LY294002, the apoptosis of DRG cells in the HG+CGRP+LY294002 group was markedly increased compared to that in the HG+CGRP group (*p* < 0.01).

### 3.3. Measurement of ROS, MDA, and SOD Levels in DRG Cells

**Figure 3:**
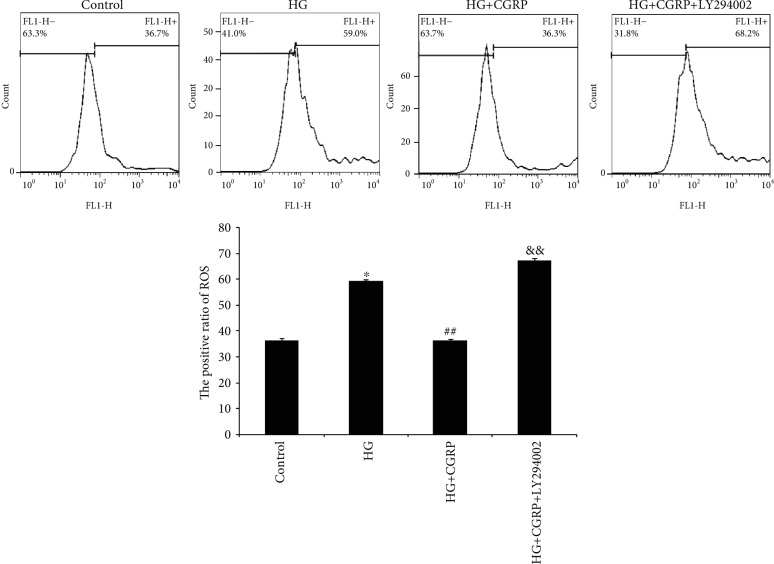
The production of ROS in DRG neurons after HG, HG+CGRP, or HG+CGRP+LY294002 treatment. The positive ratio of ROS shows the percent apoptosis cells of the total number. ^∗^*p* < 0.05, compared with the control; ^##^*p* < 0.01, compared to the HG group; ^&&^*p* < 0.01, compared to the HG+CGRP group (refer to the attachment for the high-definition image).

The ROS level in the DRG cells of the HG group was significantly elevated compared to the control group (*p* < 0.05), which had a reduced ROS level after CGRP treatment (*p* < 0.01). However, treatment with the inhibitor LY294002 continually increased the ROS level in cells in comparison to treatment with HG+CGRP (*p* < 0.01) (Figure 3).

**Figure 4:**
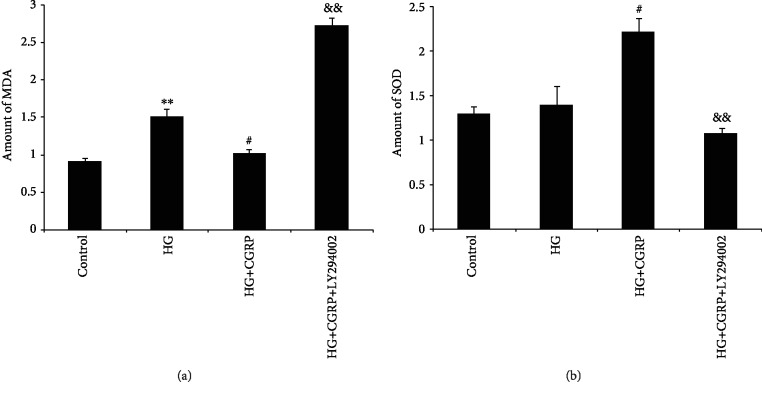
The MDA level (a) and SOD activity (b) in DRG neurons after HG, HG+CGRP, or HG+CGRP+LY294002 treatment for 48 h. ^∗∗^*p* < 0.01, compared to the control; ^#^*p* < 0.05, compared to the HG group; ^&&^*p* < 0.01, compared to the HG+CGRP group.

The MDA levels were significantly increased in the DRG cells of the HG group compared to the control group (*p* < 0.01). After treatment with CGRP, MDA levels were significantly decreased in the HG+CGRP group as compared to the HG group (*p* < 0.05). However, treatment with LY294002 led to a further increase in the level of MDA compared to treatment with HG+CGRP (*p* < 0.01) (Figure 4(a)).

There was no difference in the SOD levels of DRG cells in the HG group and the control group. However, the SOD activity was significantly lower in the HG group than in the HG+CGRP group (*p* < 0.05). The SOD activity was also markedly reduced in the HG+CGRP+LY294002 group as compared to that in the HG+CGRP group (*p* < 0.01) (Figure 4(b)).

### 3.4. Effects of CGRP on HO-1 and Nrf2 Protein Expression in DRG Cells

**Figure 5:**
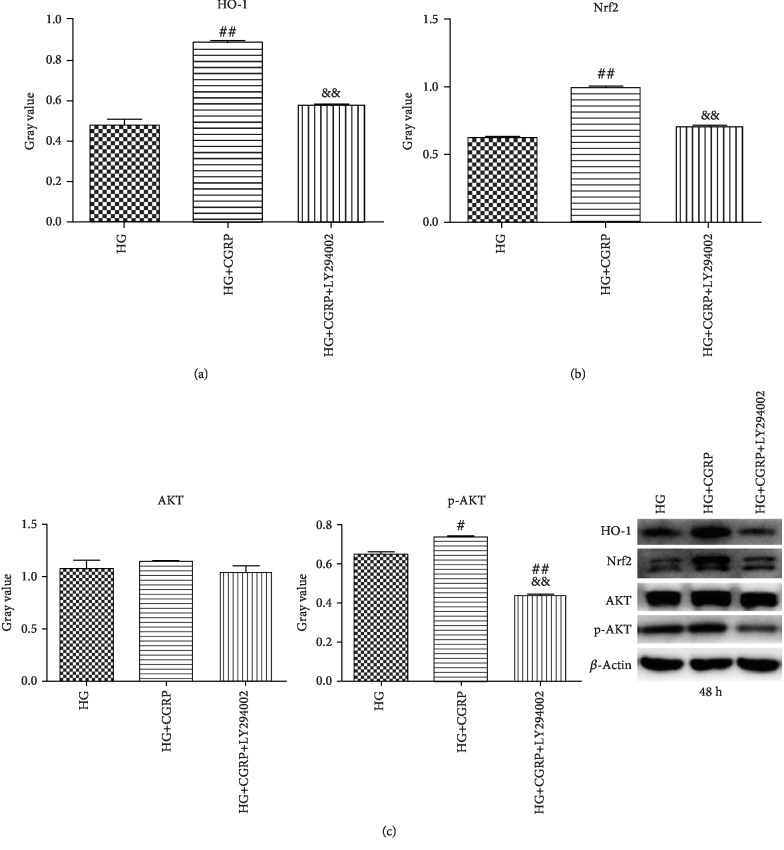
CGRP increases the protein expression of HO-1 and Nrf2 in DRG neurons. (a) The expression of HO-1 in DRG neurons with CGRP and CGRP+LY294002 treatment for 48 h and (b) the expression of Nrf2 in DRG neurons in each group determined by western blotting. (c) Phosphatidylinositol 3-kinase/AKT pathway-dependent Nrf-2 and HO-1 induction by CGRP. The expression level of AKT and the phosphorylation of AKT in DRG neurons with CGRP treatment for 48 h. ^#^*p* < 0.05, compared to the HG group; ^##^*p* < 0.01, compared to the HG group; ^&^*p* < 0.01, compared to the HG+CGRP group;^&&^*p* < 0.05, compared to the HG+CGRP group.

HO-1 is considered to be a heat-shock protein that plays an important antioxidative and antiapoptotic role in diabetes [21–23]. To explore how CGRP decreases apoptosis in DRG cells in a high-glucose culture medium, we investigated whether CGRP induces the expression of HO-1.  Figure 5 shows that the expression of HO-1 in the HG group was remarkably decreased compared to that of the HG+CGRP group. Pretreatment with LY294002 led to a markedly decreased expression of HO-1 compared to that of the HG+CGRP group (Figure 5(a)). Nrf2 is considered to be a regulator of HO-1 expression [24, 25]. Therefore, we next investigated the expression of Nrf2 in the different groups. Nrf2 expression in the HG group was markedly decreased compared to that in the HG+CGRP group. Nrf2 expression in the HG+CGRP+LY294002 group was markedly reduced compared to that in the HG+CGRP group (Figure 5(b)).

### 3.5. PI3K/AKT Signaling Is Involved in the Induction of HO-1 and Nrf2 Expression by CGRP

Finally, we wanted to elucidate the signaling pathway responsible for the induction of Nrf2 and HO-1 expression by CGRP. A previous study demonstrated that PI3K/AKT plays a crucial role in the induction of HO-1 and Nrf2 in attenuating C6, cardiomyocyte apoptosis, and renal cell damage [24, 26, 27]. Therefore, we investigated whether CGRP activates PI3 kinase and observed no difference in AKT expression among the three groups. Western blot analysis confirmed that the expression level of p-AKT was significantly increased in the HG+CGRP group as compared to the HG group. The results suggested that, as compared to the HG+CGRP group, the p-AKT expression level in the HG+CGRP+LY294002 group was downregulated by pretreatment with the PI3K/AKT inhibitor LY294002 (Figure 5(c)).

## 4. Discussion

In our present study, we have evaluated the effects of CGRP on antioxidation and antiapoptosis in a high-glucose culture model of DRG cells. Our results revealed that CGRP attenuated the apoptosis of DRG cells induced by oxidative stress injury by increasing the expression of HO-1 and Nrf2 through the PI3K/AKT pathway. To our knowledge, this is one of the first studies to report that CGRP decreases the ROS level in DRG cells in a HG-induced oxidative stress model. In recent years, increasing evidence has indicated that CGRP counters oxidative stress, improves bladder function, and is involved in the antiapoptotic process in vitro [4, 18, 28]. It has been reported that the polyol pathway increases advanced glycation end products (AGEs), hyperglycemia-induced activation of protein kinase C (PKC), and hexosamine pathway flux, which are found to participate in the hyperglycemia-induced overproduction of superoxide [6]. In a previous study, we confirmed that transcutaneous electrical nerve stimulation (TENS) improves the diabetic cystopathy (DCP) via upregulation of CGRP and cAMP. However, the role of CGRP in the pathogenesis of DCP and the mechanism of CGRP inhibiting the apoptosis of DRG cells in high-glucose conditions remain largely unclear.

DRG cells have unique anatomical and structural characteristics that make them easily vulnerable to hyperglycemic damage [29, 30]. In the first part of our study, we provide strong evidence for the role of hyperglycemia in the development of DRG damage. Our results show that DRG damage begins when the glucose concentration is 45 mmol/L at 48 h. Meanwhile, the cell viability of DRG gradually decreased with increasing concentrations of glucose and cell culture durations. The high-glucose culture condition was selected to be 45 mmol/L in accordance with previous studies [19, 20]. However, the minimum glucose concentration required for apoptosis may be different due to different experimental conditions. Evidence suggests that DRG cells cultured in a medium containing elevated (30 mmol/L) glucose concentrations undergo apoptosis in vitro [31]. The apoptotic percentage of DRG cells was increased and neurite growth was decreased in a dose-dependent manner with an increase in the glucose concentration from 0 to 300 mmol/L above the control concentrations [31]. These results were partly consistent with ours.

The concept that oxidative stress plays a key role in nerve injury in diabetes has now been confirmed [2, 7, 32, 33]. The levels of antioxidant enzymes are considered to be an important index in oxidative stress injury. To explore the state of oxidative stress, we further measured the levels of ROS, MDA, and SOD.

Our report indicates that the levels of ROS and MDA in DRGs in the HG group were significantly increased as compared to the control. The SOD activity was markedly decreased in the DRGs of the HG group compared to the control. Russel et al. suggested that, compared to the exposure at 45 mmol/L glucose, ROS production in DRG cells at 150 mmol/L glucose was significantly increased [34]. This might confirm that the oxidative stress injury of DRG cells induced by glucose was associated with the glucose concentration. It has been confirmed by previous studies that glucose-mediated oxidative stress leads to the injury of DRG cells [35, 36]. The other finding of this study is that the treatment of DRG cells with CGRP can inhibit the oxidative stress response. CGRP serves as an antioxidative mediator that participates in various diseases [18, 28]. This article showed that the level of ROS and MDA was reversed by CGRP in the HG+CGRP group compared to that in the HG group. Meanwhile, the apoptosis of DRG in the HG+CGRP group was reduced compared to the HG group. This was consistent with previous studies showing the application of antioxidants in preventing DRG neuronal death [19, 37]. But a previous study showed that CGRP cooperated with substance P to inhibit melanogenesis and induce the apoptosis of B16F10 cells. The expression of apoptotic protein was related to the concentration of CGRP [38]. So we speculated that the action of CGRP is related to its concentration and exposure time.

Nrf2 is a master regulator of redox homoeostasis and a key transcription factor mediating a wide array of antioxidant genes, such as HO-1. HO-1, the downstream target of Nrf2, was measured in our study to investigate antioxidative function. The dissociation of the Nrf2-Keap 1 complex, which is regulated via one or more upstream kinases, including PKC, PI3K/AKT, and MAPK, has recently been reviewed [39–41]. PI3K/AKT is considered to be one of the major pathways upregulating the activity of Nrf2 [42]. In our study, we discussed the role of Nrf2 and HO-1 expression and the antiapoptotic and antioxidative functions of the PI3K/AKT pathway in DRG cells induced by HG. We found that CGRP activated PI3K/AKT and increased the expression of Nrf2 and HO-1, thereby altering the activity of antioxidant enzymes, and finally, attenuating the apoptosis of DRG cells. This result indicated that the PI3K/AKT pathway partially regulates Nrf-2 expression, which is in accordance with a prior finding in H9c2 cardiomyocytes [43]. A similar mechanism was proposed in a study that reported the activation of the PI3K/AKT pathway by atorvastatin via AKT phosphorylation at position Ser473, which then mediated Nrf-2 activation [25]. Our study suggested that DRGs treated with high glucose show a marked increase in oxidative stress, as shown by excessive ROS and MDA production. However, cotreatment with CGRP significantly attenuated oxidative damage induced by high glucose, as reflected in the augmentation of SOD activity and the accompanying decrease in MDA and ROS levels.

## 5. Conclusions

Collectively, this study is the first to demonstrate that CGRP modulates oxidative stress injury in the high-glucose-induced DRG cell model via the activation of the PI3K/AKT pathway and increases the expression of Nrf2 and HO-1. CGRP may be useful as an adjuvant therapy for diabetic neuropathy in the future, owing to its antioxidative and antiapoptotic roles.

## Data Availability

The data used to support the findings of this study are available from the corresponding author upon request.

## References

[1] Bradley W. E. (1980). Diagnosis of urinary bladder dysfunction in diabetes mellitus. *Annals of Internal Medicine*.

[2] Liu G., Daneshgari F. (2014). Diabetic bladder dysfunction. *Chinese Medical Journal*.

[3] Yuan Z., Tang Z., He C., Tang W. (2015). Diabetic cystopathy: a review. *Journal of Diabetes*.

[4] Ding L., Song T., Yi C. (2013). Transcutaneous electrical nerve stimulation (TENS) improves the diabetic cytopathy (DCP) via up-regulation of CGRP and cAMP. *PLoS One*.

[5] Maritim A. C., Sanders R. A., Watkins J. B. (2003). Diabetes, oxidative stress, and antioxidants: a review. *Journal of Biochemical and Molecular Toxicology*.

[6] Brownlee M. (2005). The pathobiology of diabetic complications: a unifying mechanism. *Diabetes*.

[7] Beshay E., Carrier S. (2004). Oxidative stress plays a role in diabetes-induced bladder dysfunction in a rat model. *Urology*.

[8] Jiang Y. J., Gong D. X., Liu H. B., Yang C. M., Sun Z. X., Kong C. Z. (2008). Ability of alpha-lipoic acid to reverse the diabetic cystopathy in a rat model. *Acta Pharmacologica Sinica*.

[9] Ustuner M. C., Kabay S., Ozden H. (2010). The protective effects of vitamin E on urinary bladder apoptosis and oxidative stress in streptozotocin-induced diabetic rats. *Urology*.

[10] Burakgazi A. Z., Alsowaity B., Burakgazi Z. A., Unal D., Kelly J. J. (2012). Bladder dysfunction in peripheral neuropathies. *Muscle & Nerve*.

[11] Chen S. C., Lai C. H., Fan W. J., Peng C. W. (2013). Pudendal neuromodulation improves voiding efficiency in diabetic rats. *Neurourology and Urodynamics*.

[12] Wen Z., Hou W., Wu W. (2018). 6′-*O*-galloylpaeoniflorin attenuates cerebral ischemia reperfusion-induced neuroinflammation and oxidative stress via PI3K/Akt/Nrf2 activation. *Oxidative Medicine and Cellular Longevity*.

[13] Loboda A., Damulewicz M., Pyza E., Jozkowicz A., Dulak J. (2016). Role of Nrf2/HO-1 system in development, oxidative stress response and diseases: an evolutionarily conserved mechanism. *Cellular and Molecular Life Sciences*.

[14] Pugazhenthi S., Akhov L., Selvaraj G., Wang M., Alam J. (2007). Regulation of heme oxygenase-1 expression by demethoxy curcuminoids through Nrf2 by a PI3-kinase/Akt-mediated pathway in mouse beta-cells. *American Journal of Physiology. Endocrinology and Metabolism*.

[15] Cruz C. D. (2014). Neurotrophins in bladder function: what do we know and where do we go from here?. *Neurourology and Urodynamics*.

[16] Vega A. V., Avila G. (2010). CGRP, a vasodilator neuropeptide that stimulates neuromuscular transmission and EC coupling. *Current Vascular Pharmacology*.

[17] Sueur S., Pesant M., Rochette L., Connat J. L. (2005). Antiapoptotic effect of calcitonin gene-related peptide on oxidative stress- induced injury in H9c2 cardiomyocytes via the RAMP1/CRLR complex. *Journal of Molecular and Cellular Cardiology*.

[18] Umoh N. A., Walker R. K., Millis R. M., Al-Rubaiee M., Gangula P. R., Haddad G. E. (2014). Calcitonin gene-related peptide regulates Cardiomyocyte survival through regulation of oxidative stress by PI3K/Akt and MAPK Signaling pathways. *Annal of Clinical and Experimental Hypertension*.

[19] Vincent A. M., Stevens M. J., Backus C., Mclean L. L., Feldman E. L. (2005). Cell culture modeling to test therapies against hyperglycemia-mediated oxidative Stress and Injury. *Antioxidants & Redox Signaling*.

[20] Chen T., Li H., Yin Y., Zhang Y., Liu Z., Liu H. (2017). Interactions of Notch1 and TLR4 signaling pathways in DRG neurons of *in vivo* and *in vitro* models of diabetic neuropathy. *Scientific Reports*.

[21] Negi G., Nakkina V., Kamble P., Sharma S. S. (2015). Heme oxygenase-1, a novel target for the treatment of diabetic complications: focus on diabetic peripheral neuropathy. *Pharmacological Research*.

[22] Chen K., Gunter K., Maines M. D. (2000). Neurons overexpressing heme oxygenase-1 resist oxidative stress-mediated cell death. *Journal of Neurochemistry*.

[23] Li B., Liu S., Miao L., Cai L. (2012). Prevention of diabetic complications by activation of Nrf2: diabetic cardiomyopathy and nephropathy. *Experimental Diabetes Research*.

[24] Zhao S. M., Gao H. L., Wang Y. L., Xu Q., Guo C. Y. (2017). Attenuation of high glucose-induced rat Cardiomyocyte apoptosis by Exendin-4 via intervention of HO-1/Nrf-2 and the PI3K/AKT Signaling pathway. *The Chinese Journal of Physiology*.

[25] Ma Y., Chen Z., Zou Y., Ge J. (2014). Atorvastatin represses the angiotensin 2-induced oxidative stress and inflammatory response in dendritic cells via the PI3K/Akt/Nrf 2 pathway. *Oxidative Medicine and Cellular Longevity*.

[26] Saha S., Sadhukhan P., Sinha K., Agarwal N., Sil P. C. (2016). Mangiferin attenuates oxidative stress induced renal cell damage through activation of PI3K induced Akt and Nrf-2 mediated signaling pathways. *Biochemistry and Biophysics Reports*.

[27] Ha Y. M., Kim M. Y., Park M. K. (2012). Higenamine reduces HMGB1 during hypoxia-induced brain injury by induction of heme oxygenase-1 through PI3K/Akt/Nrf-2 signal pathways. *Apoptosis*.

[28] Schaeffer C., Vandroux D., Thomassin L., Athias P., Rochette L., Connat J. L. (2003). Calcitonin gene-related peptide partly protects cultured smooth muscle cells from apoptosis induced by an oxidative stress via activation of ERK1/2 MAPK. *Biochimica et Biophysica Acta (BBA) - Molecular Cell Research*.

[29] McHugh J. M., Mchugh W. B. (2004). Diabetes and peripheral sensory neurons what we don't know and how it can hurt us. *AACN Clinical Issues*.

[30] Devor M. (1999). Unexplained peculiarities of the dorsal root ganglion. *Pain*.

[31] Russell J. W., Sullivan K. A., Windebank A. J., Herrmann D. N., Feldman E. L. (1999). Neurons undergo apoptosis in animal and cell culture models of diabetes. *Neurobiology of Disease*.

[32] Figueroa-Romero C., Sadidi M., Feldman E. L. (2008). Mechanisms of disease: the oxidative stress theory of diabetic neuropathy. *Reviews in Endocrine & Metabolic Disorders*.

[33] Kanika N. D., Chang J., Tong Y. (2011). Oxidative stress status accompanying diabetic bladder cystopathy results in the activation of protein degradation pathways. *BJU International*.

[34] Russell J. W., Golovoy D., Vincent A. M. (2002). High glucose-induced oxidative stress and mitochondrial dysfunction in neurons. *The FASEB Journal*.

[35] Kiasalari Z., Rahmani T., Mahmoudi N., Baluchnejadmojarad T., Roghani M. (2017). Diosgenin ameliorates development of neuropathic pain in diabetic rats: involvement of oxidative stress and inflammation. *Biomedicine & Pharmacotherapy*.

[36] Vincent A. M., McLean L. L., Backus C., Feldman E. L. (2005). Short-term hyperglycemia produces oxidative damage and apoptosis in neurons. *The FASEB Journal*.

[37] Yang D., Liang X. C., Shi Y. (2016). Anti-oxidative and anti-inflammatory effects of cinnamaldehyde on protecting high glucose-induced damage in cultured dorsal root ganglion neurons of rats. *Chinese Journal of Integrative Medicine*.

[38] Zhou J., Feng J. Y., Wang Q., Shang J. (2015). Calcitonin gene-related peptide cooperates with substance P to inhibit melanogenesis and induces apoptosis of B16F10 cells. *Cytokine*.

[39] Hwang Y. P., Jeong H. G. (2010). Ginsenoside Rb1 protects against 6-hydroxydopamine-induced oxidative stress by increasing heme oxygenase-1 expression through an estrogen receptor-related PI3K/Akt/Nrf2-dependent pathway in human dopaminergic cells. *Toxicology and Applied Pharmacology*.

[40] Nguyen C. N., Kim H. E., Lee S. G. (2013). Caffeoylserotonin protects human keratinocyte HaCaT cells against H_2_O_2_-Induced Oxidative Stress and Apoptosis through Upregulation of HO-1 Expression via Activation of the PI3K/Akt/Nrf2 Pathway. *Phytotherapy Research*.

[41] Lee J. S., Surh Y. J. (2005). Nrf2 as a novel molecular target for chemoprevention. *Cancer Letters*.

[42] Basak P., Sadhukhan P., Sarkar P., Sil P. C. (2017). Perspectives of the Nrf-2 signaling pathway in cancer progression and therapy. *Toxicology Reports*.

[43] Liu S. X., Zhang Y., Wang Y. F. (2012). Upregulation of heme oxygenase-1 expression by hydroxysafflor yellow A conferring protection from anoxia/reoxygenation-induced apoptosis in H9c2 cardiomyocytes. *International Journal of Cardiology*.

